# Suppression of CRLF1 promotes the chondrogenic differentiation of bone marrow-derived mesenchymal stem and protects cartilage tissue from damage in osteoarthritis via activation of miR-320

**DOI:** 10.1186/s10020-021-00369-1

**Published:** 2021-09-22

**Authors:** Hao Xu, Changrong Ding, Cuicui Guo, Shuai Xiang, Yingzhen Wang, Bing Luo, Hongfei Xiang

**Affiliations:** 1 Department of Joint Surgery, The Affiliated Hospital of Qingdao University, Qingdao University, Qingdao, 266000 China; 2 Department of ECG Diagnosis, The Affiliated Hospital of Qingdao University, Qingdao, 266000 China; 3 Department of Sports Medicine, the Affiliated Hospital of Qingdao University, Qingdao, 266003 China; 4 Department of Pathogenic Biology, School of Basic Medicine, Qingdao University, Qingdao, 266000 China; 5 Department of Orthopedics, School of Basic Medicine, Qingdao University, Qingdao, 266000 China

**Keywords:** CRLF1, miR-320, Chondrogenic differentiation, Bone marrow-derived mesenchymal stem, Osteoarthritis

## Abstract

**Background:**

Osteoarthritis (OA) is the most prevalent chronic joint disease, and is hard to be cured at present. Cytokine receptor-like factor 1 (CRLF1) has been identified as an upregulated gene in OA cartilage. However, the precise identities and functions of CRLF1 in OA progression have remained to be fully elucidated.

**Methods:**

We used a murine model of injury-induced OA (destabilization of medial meniscus, DMM) and BMSCs to investigate the specific biological functions and mechanisms of CRLF1.

**Results:**

We found that CRLF1 was significantly increased in the DMM surgery-induced OA model and was down-regulated during chondrogenic differentiation of BMSCs. Luciferase reporter assays showed that CRLF1 was a direct target of miR-320 in BMSCs. miR-320 can reverse the effect of CRLF1 on cell proliferation, apoptosis and chondrogenic differentiation of BMSCs. Furthermore, knockdown of CRLF1 or over-expression of miR-320 can inhibit the apoptosis of primary chondrocytes.

**Conclusion:**

Suppression of CRLF1 promotes the chondrogenic differentiation of BMSCs and protects cartilage tissue from damage in osteoarthritis via activation of miR-320.

## Background

Osteoarthritis (OA) is a common musculoskeletal disease characterized by bone-cartilage homeostasis changes, leading to progressive degeneration of the synovial joint (Pereira et al. 2011). Currently, there is no effective treatment for OA other than pain relief. In recent years, cell therapy is being developed as a new method for the treatment of osteoarthritis (Neogi 2013). Bone marrow mesenchymal stem cells (BMSCs) have the potential to differentiate into chondrocytes or osteoblasts, and their anti-inflammatory properties (Motavaf et al. 2016), are considered to be the best-grafted cells for the treatment of OA (Polymeri et al. 2016). Unfortunately, because of variable amounts of cartilage tissue development and low functional properties of the repair tissue produced, the findings of BMSC-based therapies have been suboptimal so far (Im 2017). Thus, a better understanding of the molecular mechanisms that control the chondrogenesis of BMSCs is needed to promote the generation of hyaline cartilage, which resembles native cartilage (Kagami et al. 2014).

Chondrogenesis is a complex process in which chondrogenic differentiation of MSCs is profoundly affected by the extracellular microenvironment and various growth factors (de Vries-van Melle et al. 2014; Yu et al. 2012). Cytokine receptor-like factor 1 (CRLF1) is a soluble protein that is homologous with type I cytokine receptors. CRLF1 is reportedly highly expressed in damaged human knee osteoarthritic cartilage (Tew et al. 2007) and involved in osteoarthritis downstream of TGF-beta (Tsuritani et al. 2010). However, the precise identities and functions of CRLF1 in OA progression have remained to be fully elucidated. In this study, we attempted to investigate the expression of CRLF1 in cartilage tissues in mice with OA and explored the roles of CRLF1 in cell proliferation and chondrogenic differentiation of BMSCs. Furthermore, the possible molecular mechanism was investigated simultaneously and thus may be a useful therapeutic target.

## Materials and methods

### Experimental animals

Eight weeks old male C57BL/6 mice were bought from Beijing Experimental Animal Center and kept at 25 ± 3 °C in a room equipped with a 12-h:12-h light/dark cycle switch. All animal experiments have been approved by the Animal Ethical Committee of Qingdao University. Forty mice were randomly assigned into two groups: Sham surgery without any treatment (Sham, *n* = 10) and performing the destabilization of medial meniscus (OA, *n* = 30) surgery for 8 weeks.

### DMM surgery model

The DMM surgery-induced OA mice model as previously described. Briefly, the mice were anesthetized with an intraperitoneal injection (i.p.) of pentobarbital (50 mg/kg body weight), and the ventral portion of the right knee was unhaired using depilatory paste and sterilized with an antiseptic solution. The right lower limb knee joint and its surroundings shall be covered with skin, sterilized and covered with a sterile surgical towel. The medial incision of the right knee patella shall be made in dissecting microscope. The patella shall be turned to the lateral side to expose the medial meniscus tibial ligament. The ligament shall be cut off under the microscope to avoid damage to articular cartilage. The complete transection of the ligament shall be confirmed by the passive displacement of the medial semilunar plate. Reposition patella, suture joint capsule and skin. Sham operation was performed by opening and exposing the structures of the right knee and then closing the skin incision without manipulating joint tissues. After the operation, the experimental animals were kept in groups and cages, and their free activities were not restricted. The following tests were performed 8 weeks after the operation.

### Isolation of OA chondrocytes

Chondrocytes were isolated from the femoral condyles and tibial plateaus of OA mice (8 weeks after OA induction). The primary chondrocytes were isolated using 0.1% collagenase (Gibco) as described elsewhere(Yang et al. 2019). Cells were cultured in DMEM (Gibco) containing 5% FBS, 1% penicillin/streptomycin (Gibco).

### Histology

Knee-joint tissues were removed and fixed overnight in 4% paraformaldehyde at 4 °C. Then, the fixed femoral condyles were decalcified in 10% EDTA (pH 7.4) for 7 days at 37 °C and then dehydrated by different grade ethanol (70%, 80%, 90%, 95%, and 100% ethanol in water). The dehydrated specimens were embedded in paraffin. Furthermore, the 5 μm thickness and stained with safranin O/fast green (Zhou et al. 2020). The severity of OA lesions was classified by two independent researchers, according to the Osteoarthritis Research Society International (OARSI) cartilage assessment system (Chen et al. 2017).

### Establishment of mesenchymal stem cells and identification of BMSCs

BMSCs were established as previously described (Zhang and He 2014). Briefly, after the mice were anesthetized, the tibia of mice in the Sham group and the OA group was separated and obtained. The tibia was dissected in the ultra-clean table, and the ester ends of both sides of the bones were cut off, and the culture medium was inhaled with a 1 mL syringe. Bone marrow was blown out and suspended in α-Modified Eagle’s Medium (α-MEM) containing 10% FBS and 1% penicillin–streptomycin and incubated in a 5% CO_2_ humidified atmosphere at 37 °C. After 24 h, the suspension cells were discarded and the fresh culture medium was replaced, the medium was changed once every 3 days. The passage 3 cells were characterized by flow cytometry analysis (FCA) using several positive (CD29, CD44, and CD90) and negative (CD45RB and CD79a) MSC surface markers. Cells from passage 3 were used in the experiments.

### Chondrogenic differentiation induction

The third-generation of BMSCs were resuspended in 0.5 ml of cartilage-inducing medium consisting of high glucose (4.5 mg/mL) DMEM with 100 μg/mL of sodium pyruvate (Gibco), 100 nM dexamethasone (Sigma), 10 mM ascorbic acid-2-phosphate (Sigma), 40 μg/ml L-proline (Sigma), 1% ITS (Sigma), 1% penicillin/streptomycin (Gibco) and 10 ng/mL Transforming Growth Factor β1 (R&D Systems). The medium was changed every 2–3 days depending on the color of the medium and chondrogenic pellets were harvested after 7, 14, 21, and 28 days.

### Alcian blue staining

The cell pellets were fixed in 4% paraformaldehyde before being embedded in paraffin and sectioned at 5-μm thickness. The sections were stained with Alcian blue solution (PH2.5) for 30 min before being washed three times with PBS. Under a microscope, images of the stained cells were obtained.

### Immunohistochemistry

BMSCs were maintained in a chondrogenic differentiation induction medium for 28 days, the harvested pellets were fixed with 4% paraformaldehyde for 72 h, embedded in paraffin, sectioned at 5-μm thickness. The sections were rehydrated, blocked, and incubated with primary anti-Col2a1 (1:200, Abcam) at 4 °C overnight, and the fluorescent secondary antibody was incubated the next day. The nuclei were stained with DAPI, and the cells were sealed and observed under a microscope.

### Dual-Luciferase reporter assay

BMSCs were co-transfected with WT-CRLF1 or MUT-CRLF1 and miR-320/miR-125a-3p mimics or inhibitor, then cultured in an incubator for a further 48 h. Cells were collected, and double luciferase activity was detected in each group according to the instructions of the Dual-Luciferase Reporter Assay System (Promega). The outcomes were quantified in each well as the proportion of firefly luciferase/renilla luciferase activity.

### CCK8 assay

For the cell proliferation test, a CCK8 Kit (Solarbio, China) was used. In 96-well plates, 1 × 10^4^/well cells were inoculated following different treatments. CCK8 reagent was added into wells, and cell proliferation was detected at 24 h by measuring the absorbance at 450 nm with a microplate reader.

### Real-time quantified PCR (qRT-PCR)

BMSCs were harvested after transfection 72 h. Total RNA was extracted from cells with Trizol reagent (Takara, Japan). Reverse transcription was performed according to the reverse transcription kit. As previously described, qRT-PCR was performed using the QuantiTect SYBR Green PCR kit (Qiagen) on a Real-Time PCR Detection System (Bio-Rad).

### Western blot assay

Protein in tissues (sham and OA) and cells (different groups) were extracted using RIPA (Solarbio). Protein concentrations were determined by the BCA protein assay kit. Equal amounts of protein were taken from each sample, and transferring was performed with 10% SDS-PAGE and then transferred onto nitrocellulose filter (Millipore, USA), and probed with GAPDH (1:5000, Cell Signaling), Bax (1:1000, Cell Signaling), Bcl-2 (1:1000, Cell Signaling), cleaved caspase3 (1:1000, Cell Signaling), CRLF1 (1:1000, Abcam), Sox9 (1:1000, Abcam), ACAN (1:1000, Abcam), Col2a1 (1:1000, Abcam), MMP13 (1:1000, Abcam) and collagen X (1:1000, Abcam) antibody followed by appropriate secondary antibody. Proteins were detected by chemiluminescence (Millipore).

### Statistical analysis

The statistical results from three independent experiments with three technical replications were displayed as mean ± standard error means (SEM). T-test was utilized for comparisons between two groups, and the comparison in multiple groups was analyzed by one-way analysis of variance (ANOVA) followed by Tukey’s test. P values less than 0.05 were considered to be statistically different.

## Results

### CRLF1 expression was higher in the DMM surgery-induced OA mice model

**Fig. 1:**
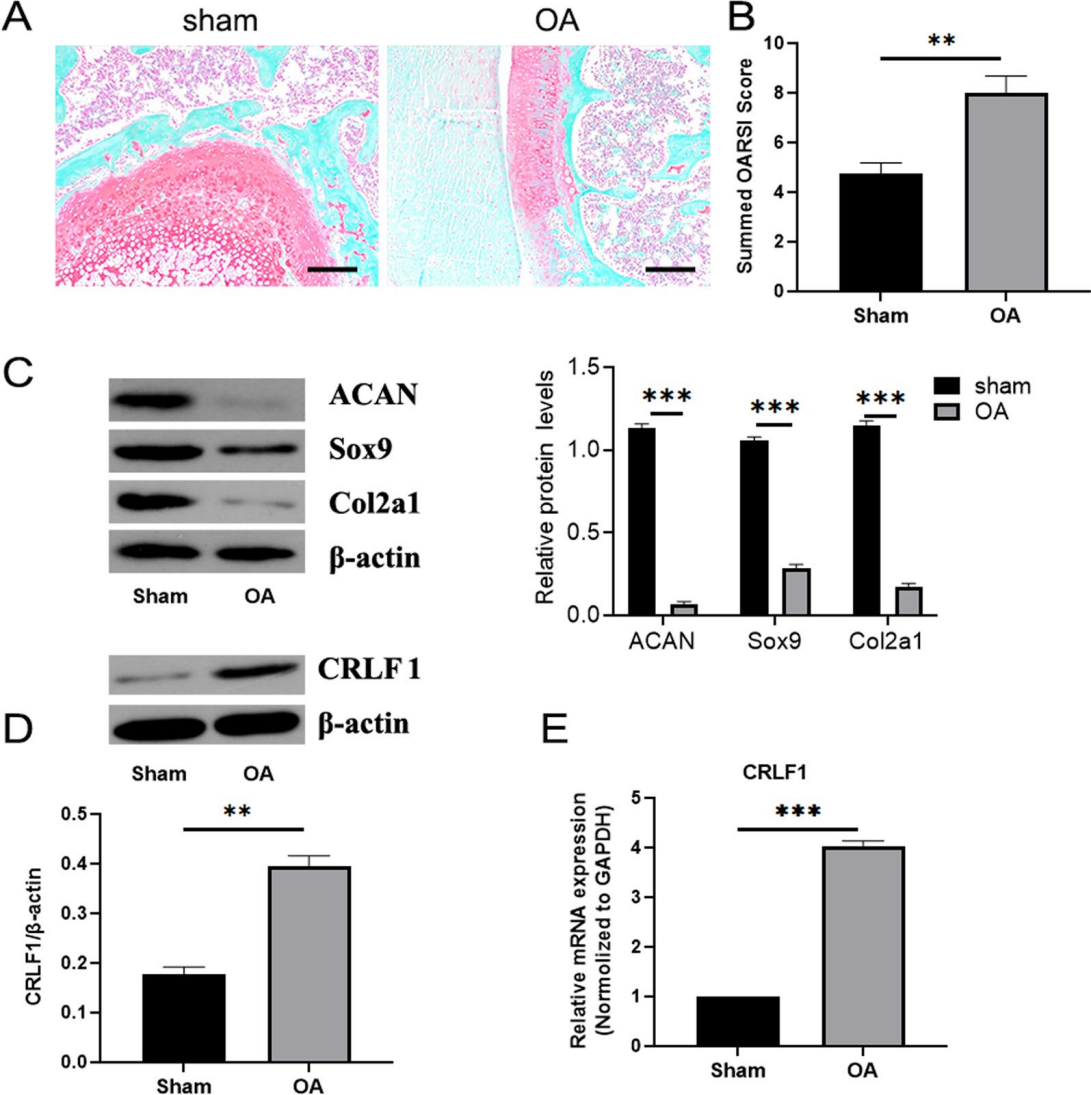
CRLF1 expression increases in DMM surgery-induced OA mice model. **A** Paraffin sections of sham and OA cartilage were processed for Safranin-O staining (scale bar, 100 μm), and **B** quantification of histology by OARSI score based on safranin O staining. **C** Protein expression levels of ACAN, Sox9, Col2a1 was determined using western blotting. **D** The mRNA and **E** protein expression levels of CRLF1 were determined using RT-qPCR and western blotting, respectively. Data are expressed as mean ± SEM and *P < 0.05, **P < 0.01, ***P < 0.01

Eight weeks after the model was established, safranine O/solid green staining was performed, and the articular anatomical structure was divided into zones. Compared with the Sham group, the articular cartilage on the medial tibial plateau surface of the knee joint was irregular, safranine O staining was reduced, and cartilage erosion and destruction in the load-bearing area reached calcified cartilage layer, accounting for about 50% of the articular surface of the medial femoral condyle in OA group (Fig. 1A). The progression of OA in the mouse DMM model was evaluated using the OARSI score (the higher the score, the more significant the articular cartilage degeneration). The OARSI score results indicated a significant increase in the OA group compared to the sham group (Fig. 1B). According to the WB results, the expression of Aggrecan (ACAN), Sry-box 9 (SOX9), Collagen types II (Col2a1) were markedly decreased in the OA group compared to the sham group (Fig. 1C). As shown in Fig. 1D, E, the expression of CRLF1 was significantly increased in DMM surgery-induced OA.

### CRLF1 was down-regulated during chondrogenic differentiation of BMSCs

**Fig. 2:**
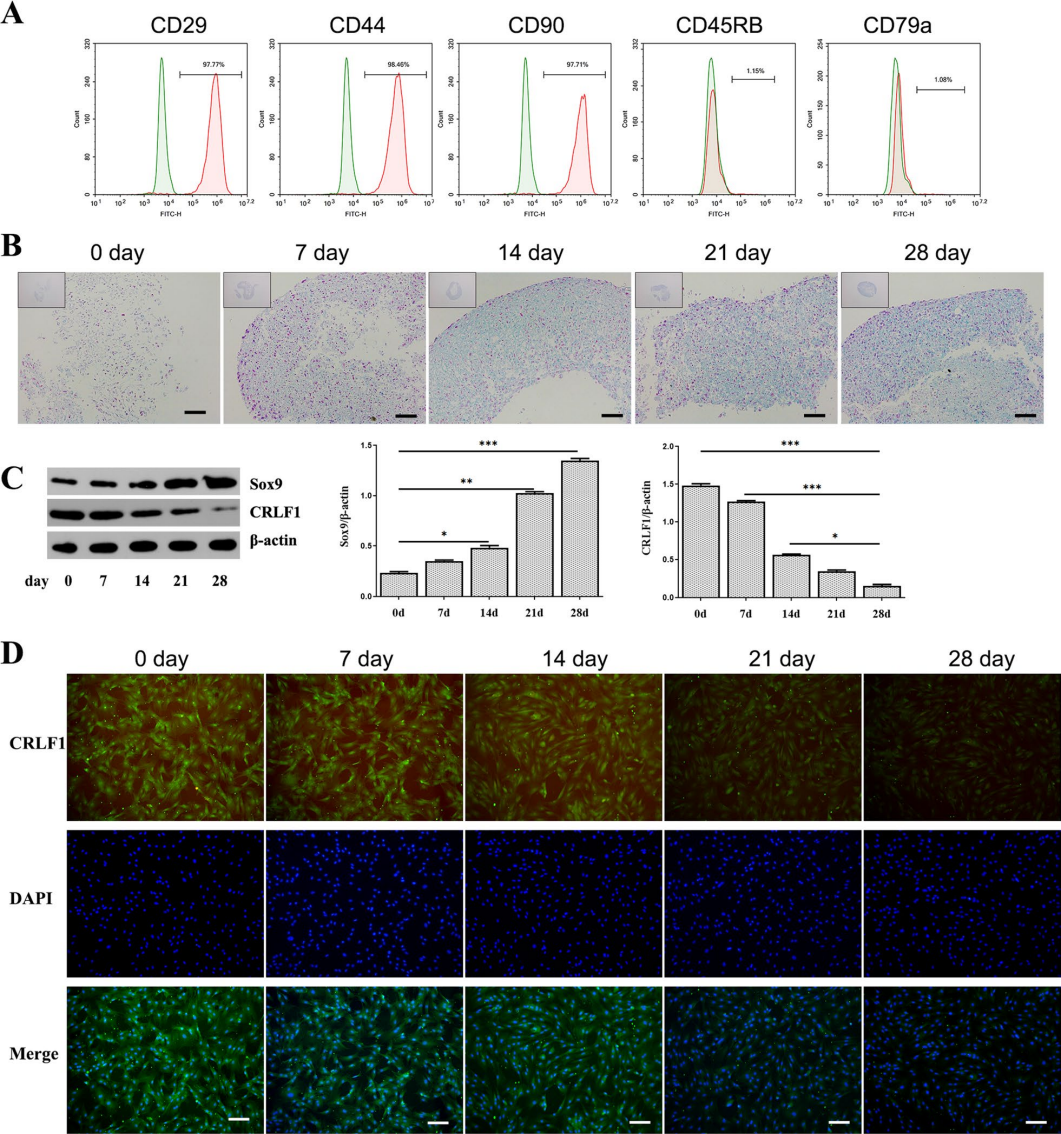
Expression levels of CRLF1 during chondrogenic differentiation of BMSCs. **A** Flow cytometric analysis of BMSC surface markers. **B** The deposition of cartilage matrix proteoglycans was determined by Alcian blue staining, nuclei were counterstained by nuclear fast red (scale bar, 100 μm). **C** Expression of Sox9 and CRLF1 was examined by WB. **D** Immunofluorescence staining for CRLF1 (green), nuclear stain DAPI (blue), and Merge for green and blue (scale bar, 100 μm); Data are expressed as mean ± SEM and *P < 0.05, **P < 0.01, ***P < 0.01

To identify the function of CRLF1 during the chondrogenic differentiation of BMSCs, BMSCs were cultured in chondrogenic media using the pellet culture system for 28 days. First, the characterization of BMSCs was analyzed by flow cytometry. The results showed that the cells were positive for CD29, CD44, CD90 and negative for CD45RB, CD79a (Fig. 2A). Alcian blue staining showed that glycosaminoglycan contents were gradually increased with the prolonged chondrogenic differentiation (Fig. 2B). In addition, the level of Sox9 was increased dramatically in BMSCs on day 28 after the induction of chondrogenic differentiation compared with day 0 (Fig. 2C). The fluorescent staining of CRLF1 further showed its distribution changes in BMSCs with chondrogenic differentiation (Fig. 2E).

### Overexpression of CRLF1 inhibited BMSCs viability, induced apoptosis and suppressed chondrogenic differentiation

**Fig. 3:**
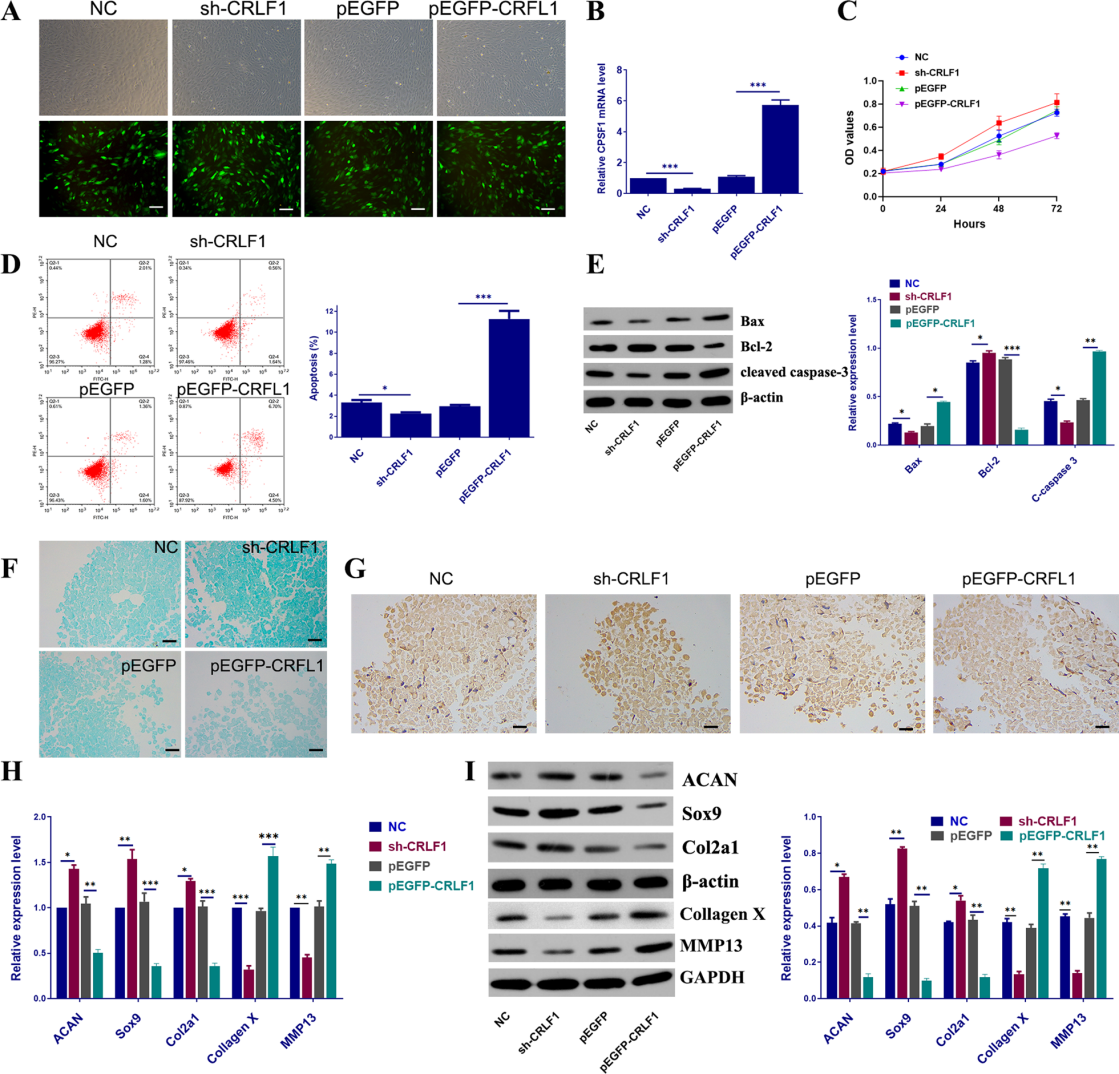
Overexpression of CRLF1 inhibited BMSCs viability, induced apoptosis and suppressed cell differentiation. **A** Infection efficiency was determined by microscopic examination of the GFP-expressing cells (scale bar, 100 μm). **B** The CRLF1 expression in different transfected BMSCs. **C** The BMSCs viability of different transfected groups. **D** Flow cytometric analysis of BMSCs apoptosis of different transfected groups. **E** The expression of apoptosis-related proteins in different transfected groups. **F** The deposition of cartilage matrix proteoglycans was determined by Alcian blue staining (scale bar, 50 μm). **G** IHC results of Col2a1 expression in different transfected groups (scale bar, 50 μm). **H** qPCR showed the relative quantification of ACAN, Sox9, Col2a1, collagen X and MMP13 mRNA levels in each group. **I** Protein expressions of ACAN, Sox9, Col2a1, collagen X and MMP13were evaluated by western blot. Data are expressed as mean ± SEM and *P < 0.05, **P < 0.01, ***P < 0.01

To investigate the function of CRLF1 in the chondrogenic differentiation of BMSCs, BMSCs were transfected with sh-CRLF1 and pEGFP-CRLF1. The BMSCs' morphology was analyzed under a fluorescence microscope after 48 h of virus infection, and the intensity of fluorescence infection confirmed that the BMSCs had been successfully infected with lentivirus (Fig. 3A). According to the qRT-PCR findings, CRLF1 expression was significantly higher in the pEGFP-CRLF1 group compared to the pEGFP group and significantly lower in the sh-CRLF1 group compared to the NC group, suggesting that transfection was successful for subsequent experiments (Fig. 3B). Using the CCK8 assay, we found that the pEGFP-CRLF1 group significantly inhibited the viability of cells, while the sh-CRLF1 group significantly promoted the viability of cells (Fig. 3C). Flow cytometry analysis revealed that pEGFP-CRLF1 enhanced BMSCs apoptosis level as compared to the pEGFP (Fig. 3D) via regulating apoptosis-related proteins (decreasing Bcl-2 expression and increasing the expression of Bax and cleaved caspase 3) (Fig. 3E). In addition, after chondrogenic differentiation for 28 days, compared with the NC group, Alcian blue staining showed that glycosaminoglycan contents in the sh-CRLF1 group were increased (Fig. 3F), and the mRNA and protein expression levels of ACAN, Sox9, Col2a1 were markedly increased, while that of collagen X and MMP13 decreased in the sh-CRLF1 group (Fig. 3G–I).

### CRLF1 3′-UTR is the direct target of miR-320 in BMSCs

**Fig. 4:**
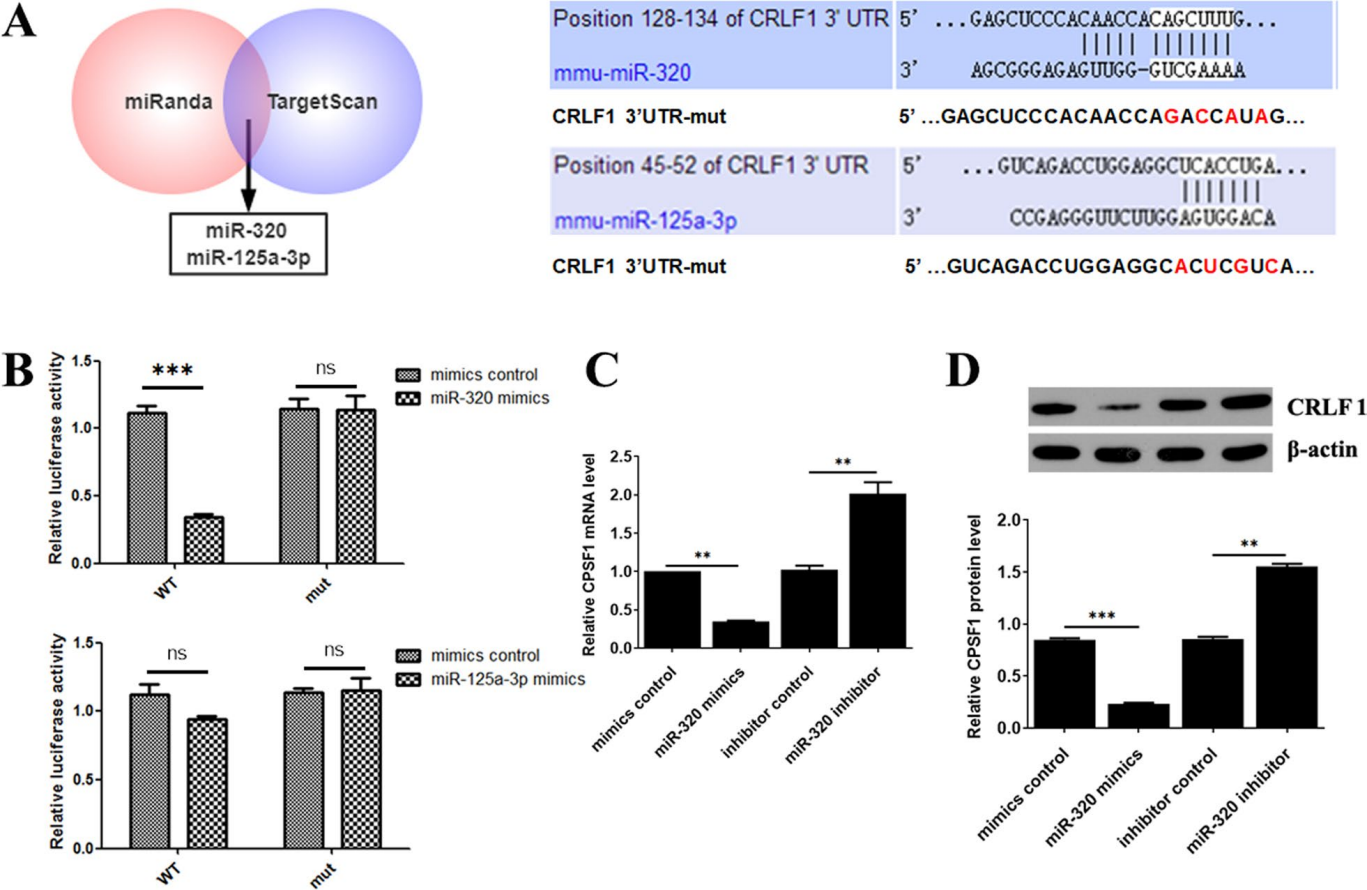
CRLF1 3′-UTR is the direct target of miR-320 in BMSCs. **A** Target sequence in the 3'-UTR of CRLF1 predicted to bind miRNA-320 and miR-125a-3p. **B** Luciferase activity of BMSCs co-transfected with WT or Mut CRLF1 3’-UTR and miRNA-320/miR-125a-3p mimic or inhibitor. **C** RT-qPCR and **D** WB measured the effect of miRNA-320 mimic or inhibitor on CRLF1 expression. ∗*p* < 0.05, ∗∗*p* < 0.01, ∗∗∗*p* < 0.001

**Fig. 5:**
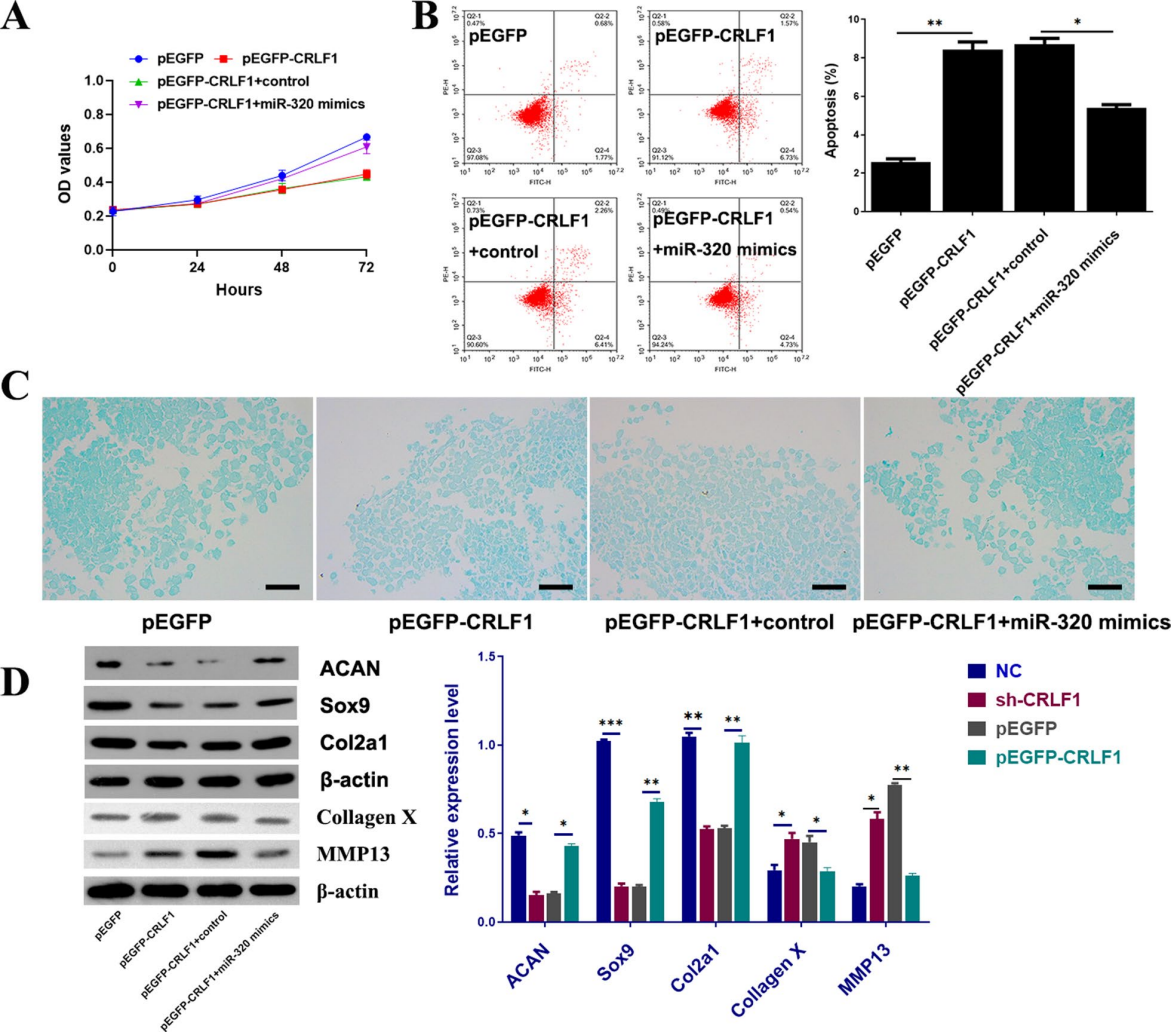
miR-320 could reverse the effect of CRLF1 on cell proliferation, apoptosis and chondrogenic differentiation of BMSCs. **A** Cell viability was estimated in BMSCs co-transfected with pEGFP-CRLF1and miR-320 mimic. **B** Apoptosis was examined by flow cytometric analysis using Annexin V/PI assay. **C** The deposition of cartilage matrix proteoglycans was determined by Alcian blue staining (scale bar, 50 μm). **D** Protein expressions of ACAN, Sox9, Col2a1, collagen X and MMP13 were analyzed by western blot. ∗*p* < 0.05, ∗∗*p* < 0.01, ∗∗∗*p* < 0.001

The important role of CRLF1 in BMSCs motivated us to identify the target genes that were directly regulated by CRLF1. As illustrated in Fig. 4A, miR-320 and miR-125a-3p directly target a potential binding site in the 3’UTR of CRLF1 according to the findings of the miRanda and TargetScan prediction algorithms. Next, to demonstrate the regulation of CRLF1 by miR-320 and miR-125a-3p, luciferase reporters were developed using the wild-type targeting sequences (CRLF1-WT) and the mutated CRLF1 3’UTRs. The dual-luciferase reporter gene activity assay further confirmed that CRLF1 was a target of miR-320, not miR-125a-3p (Fig. 4B). Next, western blotting and RT-qPCR were carried out to investigate the effects of miR-320 on CRLF1 expression. It was demonstrated that CRLF1 expression was suppressed by a miR-320 mimic, and elevated by miR-320 inhibitor in BMSCs (Fig. 5C, D). These findings suggested that CRLF1 was a direct target of miR-320 in BMSCs.

### miR-320 can reverse the effect of CRLF1 on cell proliferation, apoptosis and chondrogenic differentiation of BMSCs

BMSCs were co-transfected with pEGFP-CRLF1 and miR-320 mimic to further explore the effect of miR-320 in BMSCs with overexpression of CRLF1. After chondrogenic differentiation, using the CCK-8 assay, we found that overexpressing miR-320 significantly promoted proliferation and inhibited apoptosis induced by the CRLF1 overexpression (Fig. 5A, B). Alcian blue staining results suggested that the deposition of proteoglycans in the pEGFP-CRLF1 + miR-320 mimics group was more than that of the CRLF1 overexpression group (Fig. 5C). As Fig. 5D showed, the protein levels of Aggrecan, SOX9 and COL2A1 were remarkably elevated, while that of collagen X and MMP13 decreased in the pEGFP-CRLF1 + miR-320 mimics group when compared with the CRLF1 overexpression group alone. These results demonstrated that the up-regulation of miR-320 could reverse the inhibitory effect of overexpressed CRLF1 on proteoglycan deposition as well as ACAN, Sox9, Col2a1, collagen X and MMP13expression.

### Knockdown of CRLF1 or overexpression of miR-320 can inhibit the apoptosis of primary chondrocyte

**Fig. 6:**
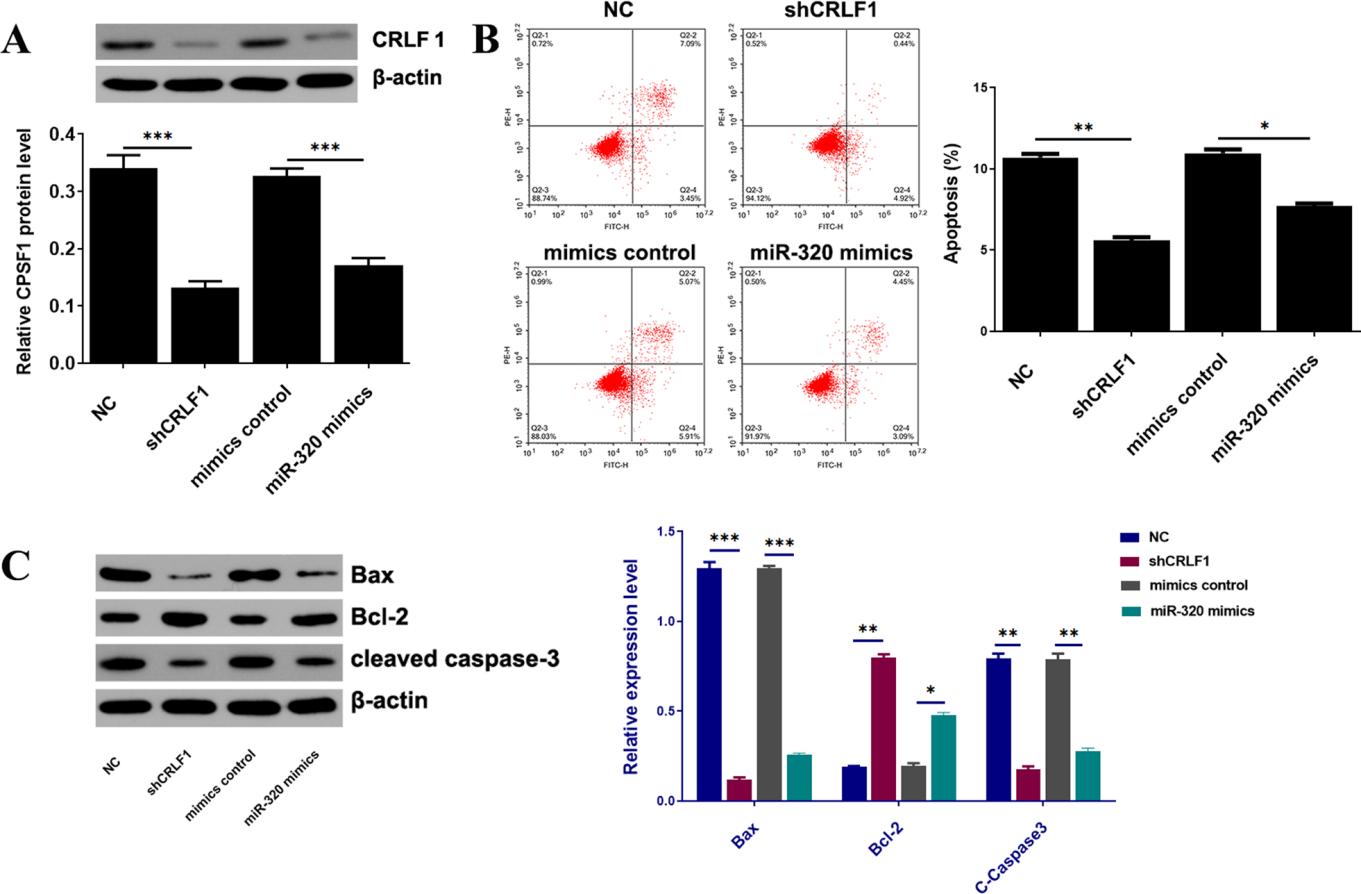
knockdown of CRLF1 or over-expression of miR-320 can inhibit the apoptosis of primary chondrocytes isolated from the articular cartilage tissues of OA mice. **A** The CRLF1 expression in chondrocytes transfected with shCRLF1, miR-320 mimics and their controls. **B** Apoptosis was examined by flow cytometric analysis using Annexin V/PI assay. **C** The expression of apoptosis-related proteins in different transfected groups. ∗*p* < 0.05, ∗∗*p* < 0.01, ∗∗∗*p* < 0.001

A substantial body of evidence exists suggesting OA significantly correlates with chondrocyte apoptosis. Therefore, the effect of CRLF1 and miR-320 on cell apoptosis was investigated in primary chondrocytes isolated from the articular cartilage tissues of OA mice. After transfection with shCRLF1 or miR-320 mimics, the apoptosis of chondrocytes was determined using flow cytometry (Fig. 6B) and the Western blotting analysis of CRLF1, cleaved caspase-3, Bax and Bcl2 (Fig. 6A, C). The above findings suggested that the knockdown of CRLF1 or overexpression of miR-320 can inhibit the apoptosis of primary chondrocytes isolated from OA articular cartilage tissues in mice.

## Discussion

While OA is a widespread and commonly occurring disease in clinical practice, there is an increasing clinical and basic OA study. However, because the occurrence of OA is the result of multi-factor activity, no substantial progress has been made in the pathogenesis and treatment of OA (Kristjansson and Honsawek 2017; Li et al. 2017). In addition, the current surgical treatment cannot effectively solve the problem of cartilage repair after injury. In recent years, with the widespread use of tissue engineering and stem cell transformation and regeneration, stem cells combined with tissue engineering to construct artificial meniscus tissue transplantation has become a research hotspot in repairing cartilage injury (Grenier et al. 2014). MSCs may be extracted from specific tissues, including the bone marrow, blood, teeth, muscle, and skin, which have particular proliferative or pluripotency advantages (Shen et al. 2019; Wu et al. 2019). BMSCs have a strong proliferative and differential capacity, shown to be the best choice for cartilage repair (Lai et al. 2015). However, the regulatory mechanism for BMSCs chondrogenic differentiation is still poorly known. In the current study, we showed that CRLF1 was significantly increased in DMM surgery-induced OA model and was down-regulated during chondrogenic differentiation of BMSCs.

CRLF1 has been reported to promote the proliferation of chondrocytes (Tsuritani et al. 2010). However, its function in the chondrogenic differentiation of BMSCs remains unclear. Therefore, we performed in vitro and in vivo experiments to explore its biological function. Our data showed that over-expression of CRLF1 inhibited BMSCs viability, induced apoptosis, and suppressed chondrogenic differentiation of BMSCs. In addition, CRLF1 is reportedly expressed in osteoblasts and chondrocytes, and its expression was elevated in SW1353 chondrosarcoma cells transduced by the key chondrogenic transcription factor SOX9 (Dagoneau et al. 2007). (Stefanovic and Stefanovic 2012). (Dagoneau et al. 2007). We also found that SOX9 was downregulated in CRLF1-overexpressing cells.

Numerous studies suggest that microRNAs (miRs) play an important role in the differentiation of stem cells by suppressing their target gene expression at the post-transcriptional level (Dong et al. 2012). In this study, the data demonstrated miR-320 directly bound to the 3’UTR of the CRLF1. miR-320 was recently found to inhibit cell proliferation and attenuate neurologic injuries after spine ischemia. A recent study showed that the expression level of MMP-13 is negatively correlated with that of miR-320, indicating that miR-320 regulates MMP-13 expression during chondrogenesis (Meng et al. 2016). Our results also suggested that miR-320 can reverse the effect of CRLF1 on cell proliferation, apoptosis, and chondrogenic differentiation of BMSCs.

## Conclusion

In the present study, we demonstrated that CRLF1 was upregulated in the DMM surgery-induced OA mice model and CRLF1 overexpression inhibited proliferation and promoted apoptosis via regulation of miR-320 in chondrogenic differentiation of BMSCs. Further investigation showed that knockdown of CRLF1 or overexpression of miR-320 can inhibit the apoptosis of primary chondrocytes isolated from OA mice articular cartilage tissues. Therefore, we provide a prospective therapeutic target for the treatment of OA.

## Data Availability

All data generated or analyzed during this study are included in this article.

## Abbreviations

ARS: Alizarin Red S
ACAN: Aggrecan
BMSCs: Bone marrow mesenchymal stem cells
CRLF1: Cytokine receptor-like factor 1
Col2a1: Collagen types II
DMM: Destabilization of medial meniscus
DMEM: Dulbecco’s modified Eagle’s medium
FBS: Fetal bovine serum
ITS: Insulin-transferrin-selenium
MMP13: Matrix metalloproteinase 13
OARSI: Osteoarthritis Research Society International
OA: Osteoarthritis
SOX9: Sry-box 9
